# Delayed Biotin Therapy in a Child with Atypical Profound Biotinidase Deficiency: Late Arrival of the Truth and a Lesson Worth Thinking

**DOI:** 10.3390/ijms241210239

**Published:** 2023-06-16

**Authors:** Shu Liu, Ye Zhang, Zhi Deng, Hui He, Xianhua Zheng, Qingshan Hong, Xianqiong Luo

**Affiliations:** 1 Pediatric Endocrinology and Inherited Metabolic Department, Guangdong Women and Children Hospital, Guangzhou 511442, China; 2 Department of Clinical Laboratory, Guangdong Women and Children Hospital, Guangzhou 511442, China; 3 Department of Radiology, Guangdong Women and Children Hospital, Guangzhou 511442, China

**Keywords:** biotinidase deficiency, *BTD* gene, novel variant, organic aciduria, biotin treatment

## Abstract

Biotinidase (BTD) deficiency (OMIM 253260) is an autosomal recessively inherited metabolic disorder resulting from deficient activity of the BTD enzyme, which can cleave and release biotin from a variety of biotin-dependent carboxylases, and is therefore recognized as a tool to recycle biotin. Being a condition caused by variations on *BTD* gene with a consequence of free biotin shortage, BTD deficiency may impair the activity of biotin-dependent carboxylases, and thus bring about a buildup of potentially toxic compounds in the body, primarily 3-hydroxyisovaleryl-carnitine in plasma as well as 3-hydroxyisovaleric acid in urine. The phenotype of BTD deficiency may vary dramatically, from asymptomatic adults to severe neurological anomalies, even death in infancy. In the present study, we reported on a 5-month-old boy, whose parents sought for medical consultation in our clinic for their son due to his loss of consciousness, repeated tetany, and motor retardation. Detailed clinical features included severe psychomotor retardation, hypotonia, as well as failure to thrive. The brain MRI at 12 months showed cerebellar hypoplasia and multiple foci of leukodystrophy. The result of antiepileptic therapy was not satisfying. During hospitalization, BTD deficiency was suggested by elevated concentration of 3-hydroxyisovaleryl-carnitine in the blood spots and 3-hydroxyisovaleric acid in the urine. The child was then diagnosed with profound BTD deficiency based on the above findings and low BTD enzyme activity. Subsequent mutational analysis revealed a novel homozygous variant, c.637_637delC (p.H213Tfs*51) in exon 4 of *BTD* gene in the proband, which was recognized as a further support to the diagnosis. Therefore, biotin treatment was started immediately, eventually with satisfactory outcomes achieved in terms of prevention of epileptic seizure, performance in deep tendon reflexes, and improvement of muscular hypotonia, but unfortunately, the therapy failed to show any evident effects on poor feeding and intellectual disability. This painful lesson suggests that newborn screening for inherited metabolic diseases is essential for early identification and treatment, which should have been performed in this case to avoid this tragedy.

## 1. Introduction

Biotinidase (BTD) deficiency (OMIM 253260), as a rare autosomal recessively inherited metabolic disorder, is closely associated with disturbances in the hydrolysis of small biotinylated peptides and biocytin, as well as the consequent failure in biotin liberation and recycling [1]. The incidence of BTD deficiency in neonates is approximately 1:61,067 worldwide, whereas the proportion of severe cases is only 1:137,401, much lower than the overall incidence [2]. Being an essential water-soluble vitamin, biotin is known as a coenzyme for multiple carboxylases, such as 3-methylcrotonyl-COA carboxylase, pyruvate carboxylase, and propionyl-COA carboxylase. Since these enzymes play critical parts in amino acid catabolism as well as glucose and fatty acid synthesis, biotin deficiency usually leads to metabolic disturbances, typically manifested as the accumulation of 3-methylcrotonyl-CoA metabolites, principally 3-hydroxyisovaleryl-carnitine (C5-OH) in blood and 3-hydroxyisovaleric acid (3-HIVA) in urine, thus resulting in neurocutaneous features and coma as the most common consequences, and even death in some of the cases [3]. The clinical manifestation of BTD varies greatly from case to case, and the patients with an enzyme activity less than 10% of the mean normal value in serum are defined as having a profound BTD deficiency, in contrast to partial BTD deficiency, a less severe condition associated with 10–30% of the normal activity. Unlike partial BTD deficiency which may have an occurrence triggered by some infectious and metabolic stresses [4], profound BTD deficiency usually shows great diversity in initial signs and symptoms, and the deficiency has severe consequences typically manifested as a multisystem disorder: developmental delay, ataxia, seizure, skin rash, sensorineural hearing loss, vision problems, etc. [5]. Meanwhile, the onset of profound BTD deficiency, though usually within the range from two months to several years of age, varies with each individual [6,7]. Hence, these features of profound BTD deficiency as described above make early identification and treatment quite challenging.

In the present study, we reported a case of a 5-month-old boy with profound BTD deficiency, who exhibited atypical clinical and metabolic features that consequently provoke a belated diagnosis and partial disability. This case provides an illustrative example for the clinical variability of the disorder, and also, the importance to raise suspicion in differential diagnosis based on the diverse clinical presentations. Our research highlights the necessity of early intervention aiming to prevent irreversible clinical manifestations by combining multiple approaches, such as biochemical, genetic, and imaging methods, in the management of this potentially treatable disease.

## 2. Results

### 2.1. Clinical Findings

A five-month-old Chinese boy with a chief complaint of “repeated tetany and motor retardation for four and a half months, and disturbance of consciousness for four hours” was admitted to our hospital through the pediatric clinic. The boy was born as the second child of healthy non-consanguineous Chinese parents at term after an uneventful pregnancy and delivery. His birth weight was registered as 2800 g (9th percentile), length 50 cm (41.7th percentile), and head circumference 35 cm (65.6th percentile). According to the history, failure to thrive, feeding problems, delayed motor, and repeated tetany were noted since the birth. Physical examination revealed severe hypotonia and psychomotor retardation. The boy had an older brother, who suffered his first grand mal epileptic seizure at one month after birth, and died at one year of age. Constrained by limited medical conditions and understanding of inherited metabolic disorders in the under-developed areas, the relevant evidence was not taken seriously.

**Figure 1:**
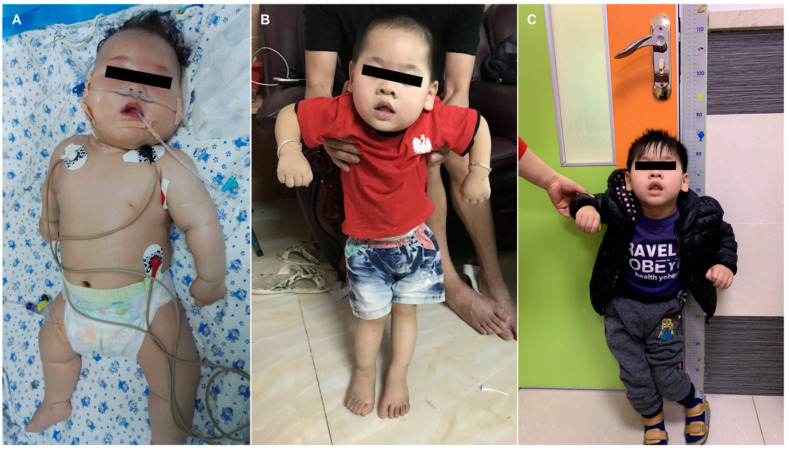
Photos taken during clinical follow-ups reveal the phenotypic features of a Chinese boy with atypical profound biotinidase deficiency. (**A**) The overall appearance of the patient indicates no abnormalities except for hypotonia as compared with normal children. Features more specific to profound biotinidase deficiency, such as typical eczematous skin rash, alopecia, conjunctivitis, or candidiasis, were not found in this patient. The photo was taken at the age of five months in our pediatric intensive care unit. (**B**,**C**) The patient was only able to stand with assistance. (**B**,**C**) were taken at the age of one year and eight months as well as three years and two months, respectively.

**Figure 2:**
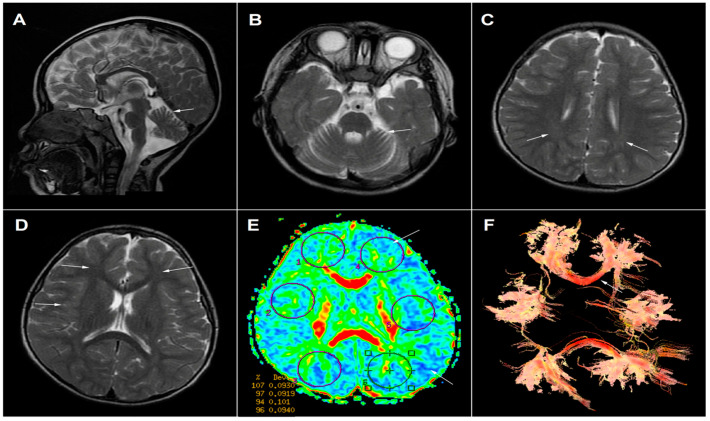
Diffusion tensor-based images of brain MRI. The T2W sagittal (**A**) and transverse (**B**) MR images of the brain reveal cerebellar hypoplasia (indicated by white arrow). T2W transverse image demonstrates hypo-intense shadow (white arrow) in the white matter of parietal cortex (**C**) as well as frontal and temporal cortices (**D**), indicating poor myelination of the cerebral white matter. Color-coded fractional anisotropy (FA) maps (**E**) were generated to depict the tracts of nervous system in the brain, with red representing the fibers running transversely from left to right, as well as green and blue respectively representing those running in the posterior-anterior and inferior-superior dimension. (**E**) reveals a mild FA reduction in left frontal cortex and occipital cortex (white arrow), and a thinning of the corpus callosum fibers within left minor forceps compared to the right part. (**F**) is a fiber tractography demonstrating that the fiber tracts originating from the corpus callosum show a random distribution and a decrease in the numbers and extension of the fibers (white arrow). Meanwhile, it also reveals a thinning of the corpus callosum fibers within left minor forceps when compared with the right part (white arrow), which is in consistent with the findings in (**E**).

On admission, the weight of the patient was 8050 g (52.4th percentile), length 68 cm (70.3th percentile) and head circumference 42.5 cm (43.7th percentile). A male baby with a poor response to external stimuli, severe hypotonia, and psychomotor retardation was noticed from the first glimpse at the patient. Neurological examination revealed nothing of focal deficits but a distinct development delay. No cutaneous anomalies were found in the patient either, such as eczematous skin rash, alopecia, or candidiasis (Figure 1A). Additionally, there were no significant abnormalities detected in routine biochemistry tests associated with liver function, kidney function, blood glucose, and blood ammonia. Arterial blood-gas analysis showed metabolic acidosis complicated with respiratory alkalosis (pH = 7.07, pCO_2_ = 23 mmol/L, HCO_3_^−^ = 6.5 mmol/L). An elevation was observed in the concentrations of serum and cerebrospinal fluid lactate—6 mmol/L and 9 mmol/L, respectively. Dried blood spot (DBS) acylcarnitine and amino acids profile measured by ultra-performance liquid chromatography–tandem mass spectrometry (UPLC-MS/MS) showed elevated levels of C5-OH-carnitine (3.345 μM/L; normal range 0.08–0.70 μM/L), propionylcarnitine (C3) (6.496 μM/L; normal range 0.4–4.0 μM/L), alanine (704.890 μM/L; normal range 126–520 μM/L), and valine (335.017 μM/L; normal range 46–230 μM/L). Urine organic acid analysis by gas chromatography mass spectroscopy (GCMS) revealed markedly increased 3-HIVA (459.27 mmol/mol creatinine; normal range 0–2.3 mmol/mol creatinine), pyruvic acid (997.31 mmol/mol creatinine; normal range 0–24.1 mmol/mol creatinine), lactate (785.69 mmol/mol creatinine; normal range 0–4.7 mmol/mol creatinine), and 3-hydroxypropionic acid (544.59 mmol/mol creatinine; normal range 0–1.1 mmol/mol creatinine), in addition to a modest elevation in 3-methylcrotonoylglycine (17.57 mmol/mol creatinine; normal range 0–1.1 mmol/mol creatinine) and methylcitrate (44.39 mmol/mol creatinine; normal range 0–1.1 mmol/mol creatinine). The clinical manifestation combined with blood acylcarnitine and the urine organic acid profile was suggestive of BTD deficiency or holocarboxylase synthetase deficiency (HLCS). Limited by imperfect medical conditions of our hospital, serum enzyme activity was determined in the other medical institutions, with the results indicating serious deficiency in biotinidase enzyme activity (0.45 nmol/min/mL serum; normal range 5.19–14.09 nmol/min/mL serum). The patient, therefore, was presumably diagnosed as having profound BTD deficiency, and oral administration with biotin was initiated at a dosage of 7.5 mg, twice daily. Meanwhile, the boy received a tailored protein-poor diet, mainly low-leucine, with a protein intake of 1.5 g/kg per day, in addition to L-carnitine supplementation (100 mg/kg per day) during his stay in the hospital. After discharge, the boy continued to take examinations in our outpatient clinic, irregularly, on and off, because of the poor compliance. In the follow-ups, the boy was monitored by plasma acylcarnitines, amino acids (mainly C5-OH), and urine organic acids (mainly 3-HIVA) every three to six months. Evaluation of his physical and mental development was conducted every six months (Figure 1B,C). The results revealed that C5-OH and 3-HIVA excretion kept fluctuating from medium to low levels (shown in Supplementary Table S1). A brain MRI at 12 months showed cerebellar hypoplasia and poor myelination of the cerebral white matter in the parietal, frontal, and temporal cortex (Figure 2). To achieve targeted therapeutic effect, further rehabilitation training, biotin (15 mg per day), and L-carnitine supplementation (50 mg−100/kg per day) were continued, paired with a tailored low-leucine diet. The dosage adjustment of protein intakes and the other drugs were performed based upon his clinical symptoms, the measurements of plasmic and uric metabolites, as well as other biochemical indexes. In the last follow-up evaluation at five years and two months old, the therapy consisted of biotin (10 mg per day) and L-carnitine supplementation (50 mg/kg per day), in combination with regular rehabilitation training. Protein restriction was mainly introduced in acute period with manifestations such as metabolic acidosis, convulsion, hyperpyrexia, or hyperammonemia attack, as a tool for dietary management, trying to offset the negative impacts from the poor compliance to the medication scheme. The comprehensive treatment was proven effective in preventing epileptic seizure, the success in drawing out brisk deep tendon reflexes, and substantial improvement in muscular hypotonia; however, it showed no evident improvement in feeding or intellectual disability. In light of these results, the previous diagnosis of BTD deficiency was, again, scrutinized by the pediatricians using molecular techniques for further verification.

### 2.2. Genetic Studies

**Figure 3:**
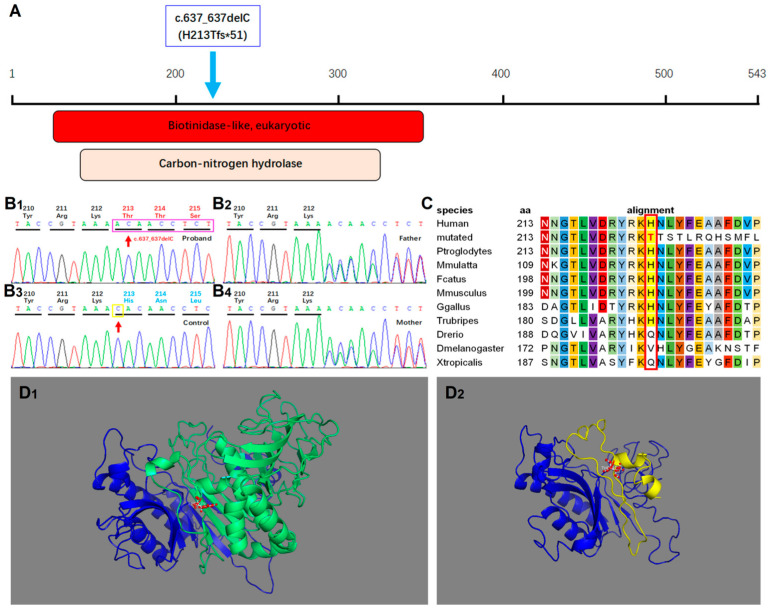
Identification of the variant in *BTD* gene. (**A**) Graphical view of protein domain and structure of *BTD*. The red box represents the Biotinidase-like (eukaryotic) domain (IPR012101); and the light apricot box represents the Carbon-nitrogen hydrolase superfamily (IPR036526). The homozygous variant of c.637_637delC (p.H213Tfs*51) is marked by wathet blue arrow. (**B1**–**B4**) Sequence chromatograms of the *BTD* variant. (**B1**–**B4**) respectively represent the variant sequences of c.637_637delC in the patient, his father, mother and normal control. The variant and wild-type sites are indicated by arrows. (**C**) BLAST comparison of the sequence around amino acids 213 in orthologs of *BTD* among species. The block and colored characters indicate the amino acid highly conserved among species, and the white shading indicates inconsistent residues. (**D1**,**D2**) Structural difference between wild-type and C-terminal frameshift *BTD* proteins. Function predictions of the mutant protein and the wild-type (full-length) *BTD* were carried out. The full length wild-type protein is displayed in the left panel (**D1**), and the mutant protein (p.H213Tfs*51) in the right panel (**D2**). In the structural model of the mutant *BTD* proteins (**D2**), the frameshift portion resulting from the p.H213Tfs*51 variant is highlighted in yellow, corresponding to the wild-type portion depicted in green (**D1**). It is clear that the variant disrupts the original structure and integrity of the *BTD* protein.

The presence of a homozygous variant, c.637_637delC (p.H213Tfs*51) in exons 4 of *BTD*, was revealed by sequencing all the exons and flanking intron regions of the *BTD* gene (Figure 3A,B). Neither the published literature nor dbSNP, ExAC, gnomAD, ClinVar, or HGMD databases have ever reported this variant. Being heterozygote carriers for the deletion, the parents did not show any symptoms of the disorder. As is known, this variant was computationally predicted to be deleterious and morbigenous by CADD, PROVEAN, and Mutation Taster. Results of bioinformatic analysis also strongly suggested that it was a disease-causing variant. By contrast, this variant was not detected in any of the 100 Chinese healthy subjects in the control group. Therefore, all abovementioned results ruled out the possibility of a polymorphism and suggested its novel and rare occurrence. The novel homozygous variant c.637_637delC (p.H213Tfs*51) was identified as a pathogenic factor according to the Sherloc/ACMG criteria: the variant was not recorded in any of the existing population databases, and was assumed to be closely associated with a highly conserved amino acid, and also, to be disease-causing according to in silico algorithms used for pathogenicity prediction [8]. Since the p.H213Tfs*51 variant is positioned at the biotinidase-like (eukaryotic) domain of the protein, it is likely to cause a direct impairment on the catalytic activity of BTD. The functional significance of the mutated amino acid is evident considering its high evolutionary conservation from mammals to invertebrates (Figure 3C,D).

The study was approved by the Ethics Committee of Guangdong Women and Children Hospital (protocol code: LL20230517006). Informed consent was obtained from the participants for publication of this case report (including all data and images). All the procedures in this study were performed in compliance with the Declaration of Helsinki.

## 3. Discussion

Biotin, an essential B-complex vitamin, can be acquired only from dietary sources due to the impossibility of endogenous synthesis in mammals; therefore, it is classified as an essential nutrient for all the living organisms. In eukaryon, biotin acts as the coenzyme of biotin-dependent carboxylases, which are known to be able to catalyze a variety of critical reactions in multiple physiological processes, such as gluconeogenesis, amino acid catabolism, and fatty acid synthesis [9,10]. Biotinidase is an enzyme that plays a key role in biotin recycling by accelerating the release of biotin from biocytin (biotinyl-lysine), which, as a by-product of apocarboxylase-catalyzed proteolytic degradation reactions [11], can be transformed into free biotin in the existence of biotinidase by removal of a lysine group. It is known that biotin deficiency can cause malfunction of multiple carboxylases (MCC, ACC, PCC, PC), and thereby impede the process of reactions catalyzed by these enzymes, resulting in toxicity related to accumulated substrates, and eventually, various clinical manifestations [11]. BTD deficiency is a relatively rare autosomal recessive disorder that may compromise multiple systems with diversified abnormal presentations, typically, eczematous skin rash, ataxia, seizures, hypotonia, developmental retardation, vision disorders, and hearing loss, as well as some respiratory symptoms and signs. Nevertheless, these features, being nonspecific, are also found in a number of inherited metabolic diseases other than BTD deficiency, especially isolated beta-methylcrotonyl-CoA carboxylase deficiency (OMIM PS210220) and holocarboxylase synthetase deficiency (OMIM 253270), as well as other disorders such as acquired biotin deficiency, meningitis (seizures and rash), and myelopathy, thus easily leading to misdiagnosis and missed diagnosis [1,5]. The incidence of profound and partial BTD deficiency worldwide is 1: 137,401 and 109,921, respectively [2]. However, credible data that can be used to define the prevalence of BTD deficiency in China are still not available due to lack of knowledge and awareness of this disease. Based on biotinidase enzyme activity in the serum, BTD deficiency was classified into two types: profound deficiency and partial deficiency, which are defined by a biotinidase activity <10% and between 10% and 30% of the mean normal activity, respectively [12]. The most conspicuous clinical features of BTD deficiency are neurologic and cutaneous disorders, which, however, have been recognized as nonspecific manifestations that may also present in other inherited metabolic diseases. Unlike partial BTD deficiency which may only be triggered by some stressors such as infection or starvation [4], the initial clinical manifestations as well as the symptoms and signs in advanced stages of profound BTD deficiency may vary significantly, even within the same family [5]. Meanwhile, stratified by the age at which the clinical manifestation appears, profound BTD deficiency was also divided into two categories: early onset and late onset. For the type of early onset, the patients may show symptoms as early as 2 to 5 months of age, but evident clinical findings may not be observed until they are a few years old [6]. Moreover, the manifestations vary greatly in the patients of untreated early onset. The most commonly observed features involving neurologic system in untreated patients with early onset BTD deficiency are seizures and hypotonia [11,13]. Myoclonic seizure was identified as the most typical type in these patients, followed by grand mal and focal seizure, and infantile spasms were also found in some children [14]. Additionally, some children without being treated properly also exhibited symptoms and signs of spinal cord impairment, particularly myelopathy and progressive spastic paresis [15,16]. Sensorineural hearing loss [17] and eye problems [18] were also reported in these children. Respiratory problems were commonly caused by hyperventilation and laryngeal stridor, and the occurrence of apnea was also observed in some cases [19]. Plus, one death initially attributed to sudden infant death syndrome was eventually identified as being due to BTD deficiency [3]. As for late onset, a number of children were absent of symptoms until adolescence when they sought for medical consultation for sudden loss of vision, sometimes with a concurrency of progressive optic neuropathy or spastic paraparesis [20,21].

It is known that early diagnosis and prompt treatment are crucial in the management of BTD deficiency due to its nature of being detrimental to the health, and even a threat to the lives of the children. However, it is still extremely challenging for pediatricians, considering its variable clinical presentations which, not uncommonly, may lead to missed diagnosis and misdiagnosis. Therefore, for a family with a history of child death in infancy or early childhood, inherited metabolic diseases, such as BTD deficiency, need to be taken into consideration for differentiation. Since clinical findings suggesting BTD deficiency are primarily based on a clear recognition of the characteristic neurologic and cutaneous anomalies, for cases with skin rash, seizures, hypotonia, acidosis, visual and auditory abnormalities, and growth retardation, most of the time there is an unambiguous and straightforward diagnosis to make [22]. Auxiliary examinations are also helpful for diagnosis. Newborn screening for BTD deficiency, which is cost effective, does not necessarily include molecular testing. The intervention should be made simple, quick, and accurate: blood taken from newborn’s heels, and DBS acylcarnitine and amino acid profiles measured by UPLC-MS/MS, by which we are usually able to obtain the results within 3–4 days. In general, laboratory evidence is referential, particularly elevation of C3 and C5-OH in acylcarnitine profile and β-Hydroxyisovalerate in urine organic acid profile. Moreover, as a useful and non-invasive tool for detecting the abnormalities of brain structures, imageological findings are informative for confirmed diagnosis and clinical management. By scanning the brain with magnetic resonance imaging or computerized tomography, for instance, various abnormalities involving with central nervous system were revealed in many symptomatic patients with BTD deficiency [23,24]. Nevertheless, it should be noticed that these imaging manifestations are also nonspecific.

The *BTD* gene is responsible for encoding a glycosylated protein with 543 amino acids, in which a signal peptide was found critical by targeting some extracellular enzymes (UniProt P43251-1) (https://www.uniprot.org/uniprot/P43251 (accessed on 14 April 2009)). In addition to the signal peptide (residues 1–41), the encoded canonical BTD protein also has a carbon-nitrogen hydrolase (CN-hydrolase) domain (residues 72–351) contained in a large chain (residues 42–543). It is reported in the ClinVar (https://www.ncbi.nlm.nih.gov/clinvar/ (accessed on 2 August 2021)) that over 190 pathogenic and suspected pathogenic variations have been detected in the *BTD* gene, with a correlation analysis employed to analyze the genotypes and the patients’ serum biotinidase activity and to assess the pathogenicity of these variants. Therefore, to clarify the pathogenesis and achieve a better management for BTD deficiency, the pathogenic variants which may be associated with abnormal development and phenotypic changes should be put on the list of pediatricians and researchers for careful scrutiny.

In this case, the predominant presentations were acute loss of consciousness and long-term repeated tetany and motor retardation, but these features, also found in other inherited metabolic disorders, are nonspecific and common in pediatric intensive care unit and pediatric neurology clinics; therefore, they had not been used to support a confirmed diagnosis of BTD deficiency until the molecular genetic testing. In this test, we identified a novel homozygous variant, c.637_637delC (p.H213Tfs*51) in exons 4 of *BTD*, which was inherited from both of his parents. This novel variant in exon 4 may lead to the substitution of the remaining 331 C-terminus amino acid residues with 50 mutant ones, at a highly conserved position of this protein. Based on the clinical presentations, imageological manifestations, and bioinformatics study, an inference stood out from the potential pathogeneses that the disease of the proband is very likely to be attributed to the homozygous variant, which, certainly, needs to be further verified by functional experiments in the future. Unfortunately, delayed diagnosis and treatment have resulted in the poor prognosis observed in this patient. However, this orphan case gave us a valuable lesson, and deepened our knowledge of BTD deficiency: the consequences caused by a belated confirmation of the diagnosis, and perhaps the death of the patient’s older brother could have been successfully prevented by newborn screening for BTD deficiency followed by early treatment with biotin supplements. Compared with the subsequent rehabilitation treatment, newborn screening and early biotin supplements for BTD deficiency are less expensive and easier to operate. In developed countries, BTD deficiency screening, characterized by simple, quick, and accurate operation without a need for compulsory molecular testing, has proved to be cost effective [6,25,26], which has set an example for our country in implementing such policies aiming to have a wide access to BTD deficiency identification through UPLC-MS/MS on DBS.

## 4. Conclusions

In our study, we presented a comprehensive delineation of the first-reported Chinese infant patient with BTD deficiency whose condition was rooted in a novel homozygous variant c.637_637delC (p.H213Tfs*51) of the *BTD* gene. Our findings reveal that tandem mass spectrometry for neonatal screening and diagnosis of inherited metabolic diseases are critical for early diagnosis of BTD deficiency, and immediate intervention by biotin supplementation to avoid harmful sequelae and death. Further diagnostic testing and molecular genetic testing are tools for a confirmed diagnosis.

## Data Availability

All data collected or generated for analysis in this study are included in this manuscript (and its Supplementary files).

## Supplementary Materials

The supporting information can be downloaded at: https://www.mdpi.com/article/10.3390/ijms241210239/s1.

## Abbreviations

BTD	Biotinidase deficiency
C5-OH	3-hydroxyisovaleryl-carnitine
C3	Propionylcarnitine
3-HIVA	3-hydroxyisovaleric acid
UPLC-MS/MS	Ultra-performance liquid chromatography–tandem mass spectrometry
GCMS	Gas chromatography–mass spectroscopy
