# Supplementary material for: Delayed Biotin Therapy in a Child with Atypical Profound Biotinidase Deficiency: Late Arrival of the Truth and a Lesson Worth Thinking
Source: Int J Mol Sci. 2023 Jun 16;24(12):10239. doi: 10.3390/ijms241210239 (PMC10299609; doi:10.3390/ijms241210239)
Supplement: Supplementary file 1 [file ijms-24-10239-s001.zip › ijms-2418362-supplementary.pdf]

Supplementary Table S1 - Biomarkers found altered in the analyzed patient. The acylcarnitine levels were quantified by UPLC-MS/MS analysis on DBS, and the organic acid levels in urine were quantified by GCMS analysis. Reference intervals are reported in brackets. DBS: dried blood spot, UPLC-MS/MS: ultra-performance liquid chromatography-tandem mass spectrometry, GCMS: gas chromatography mass spectroscopy.

Age	DBS acylcarnitines (µM/L)		Urine organic acid (mmol/mol creatinine)					
	C5-OH (0.08-0.70)	C0 (10-58)	3-hydroxyisovaleric acid (0-2.3)	Pyruvic acid (0-24.1)	Lactate (0-4.7)	3-Methylcrotonoylglycine (0-0)	3-hydroxypropionic acid (0-1.1)	Methylcitrate (0-1.1)
5m	3.345	11.538	459.27	997.31	785.69	17.57	544.59	44.39
5m	3.060	53.889	61.04	15.51	0	6.74	2.86	3.69
1y9m	0.241	36.727	0	3.65	0	0	0	0
2y4m	0.330	36.825	0	12.82	2.43	0	0	0
3y	0.314	40.775	0	4.40	0.95	0	0.95	0
3y2m	0.382	43.074	0	9.30	0	0	2.37	0.07
3y11m	0.427	33.226	8.53	22.75	2.77	0	3.95	0
5y2m	0.437	27.274	1.14	2.78	0	0	0	0.21
